# Maternal aripiprazole exposure interacts with 7-dehydrocholesterol reductase mutations and alters embryonic neurodevelopment

**DOI:** 10.1038/s41380-019-0368-6

**Published:** 2019-02-11

**Authors:** Thiago C. Genaro-Mattos, Luke B. Allen, Allison Anderson, Keri A. Tallman, Ned A. Porter, Zeljka Korade, Károly Mirnics

**Affiliations:** 1 Munroe-Meyer Institute, University of Nebraska Medical Center, Omaha, NE USA; 2 Department of Pediatrics, University of Nebraska Medical Center, Omaha, NE USA; 3 Department of Chemistry, Vanderbilt University, Nashville, TN USA

**Keywords:** Predictive markers, Diagnostic markers

## Abstract

Mutations in both copies in the gene encoding 7-dehydrocholesterol reductase (DHCR7) cause Smith–Lemli–Opitz Syndrome (SLOS), which is characterized by a toxic elevation in 7-dehydrocholesterol (7-DHC). Aripiprazole (ARI) exposure, independent of genetic mutations, also leads to elevation of 7-DHC. We investigated the combined effect of a single-copy *Dhcr7*^*+/−*^ mutation and maternal ARI exposure on the developing offspring brain. We generated a time-pregnant mouse model where WT and *Dhcr7*^*+/*^^−^ embryos were maternally exposed to ARI or vehicle (VEH) from E12 to E19 (5 mg/kg). Levels of cholesterol, its precursors, ARI and its metabolites were measured at P0. We found that ARI and its metabolites were transported across the placenta and reached the brain of offspring. Maternal ARI exposure led to decreased viability of embryos and increased 7-DHC levels, regardless of maternal or offspring *Dhcr7* genotype. In addition, *Dhcr7*^*+/*^^−^ pups were more vulnerable to maternal ARI exposure than their WT littermates, and maternal *Dhcr7*^*+/*^^−^ genotype also exacerbated offspring response to ARI treatment. Finally, both 7-DHC levels and 7-DHC/cholesterol ratio is the highest in *Dhcr7*^*+/*^^−^ pups from *Dhcr7*^*+/*^^−^ mothers exposed to ARI, underscoring a potentially dangerous interaction between maternal *genotype*×*embryonic genotype*×*treatment*. Our findings have important clinical implications. SLOS patients should avoid drugs that increase 7-DHC levels such as ARI, trazodone and haloperidol. In addition, treatment with 7-DHC elevating substances might be potentially unsafe for the 1–1.5% of population with single-allele disruptions of the *DHCR7* gene. Finally, prenatal and parental genetic testing for *DHCR7* should be considered before prescribing sterol-interfering medications during pregnancy.

## Introduction

Proper cholesterol metabolism is essential for normal brain function. Cholesterol serves not only as a membrane component but also as a precursor for bile acids, hormones, and other biologically relevant metabolites [1, 2]. Owing to the impermeability of the blood–brain barrier to cholesterol, it needs to be synthesized in situ [3] in a sequence of complex enzymatic reactions, which uses acetyl-CoA units to make cholesterol [4]. Altered cholesterol metabolism is linked to a variety of diseases, including genetic neurodevelopmental disorders [5–8]. One of them is Smith–Lemli–Opitz Syndrome (SLOS), an inherited neurodevelopmental disorder characterized by multiple congenital malformations and defects, photosensitivity, impaired cognitive function, and behaviors characteristic of  autism spectrum disorders [7, 9].

SLOS is caused by mutations in both copies in the gene encoding the last enzyme in the cholesterol biosynthesis pathway—7-dehydrocholesterol reductase (DHCR7) (Scheme 1, supplemental material) [7, 10–12]. The most significant biochemical change seen in SLOS patients is the dramatic elevation in 7-dehydrocholesterol (7-DHC) [7, 13, 14]. This compound is highly oxidizable and toxic to cells, affecting neuronal viability, proliferation, and differentiation, thus contributing to the pathophysiology of the disorder [15–19]. It is believed that heterozygous *DHCR7* mutation carriers (parents of SLOS children) have 1–1.5% frequency in the human population, and they are considered healthy [20, 21]. However, it has been recently reported that dermal fibroblasts from heterozygous carriers show elevated 7-DHC levels when compared to cells from individuals carrying the *DHCR7*^*+/+*^ genotype [22].

In addition to genetic alterations in the cholesterol biosynthesis, many chemicals are able to disrupt different steps of the cholesterol biosynthesis pathway [23–27]. A screen of the NIH small molecule collection revealed that in cultured cells ~5% of the compounds elevate 7-DHC by inhibiting DHCR7 [24]. Interestingly, antipsychotics are among the most potent 7-DHC elevators, including aripiprazole (ARI), an atypical antipsychotic that was the medication with the highest gross sales in 2013 and 2014 in the US (http://www.drugs.com/stats/abilify). ARI increases 7-DHC levels both in vitro and in vivo [22, 23, 28] and inhibits the de novo cholesterol synthesis [29]. In addition, a recent report used primary human fibroblasts to show that the combination of the two 7-DHC-elevating mechanisms (genetic and environmental) results in much higher 7-DHC levels [22], suggesting an unwanted and potentially dangerous synergism between these mechanisms. This study revealed that an exposure to the same concentrations of ARI results in higher levels of 7-DHC in cells with a *DHCR7*^*+/*^^−^ genotype when compared to *DHCR7*^*+/+*^ cells. As a conclusion, it was suggested that cells with *DHCR7*^*+/*^^−^ genotype are more vulnerable to ARI’s unwanted side effects on cholesterol biosynthesis.

The vulnerability of individuals with a *DHCR7*^*+/*^^−^ genotype to side effects of ARI has potential clinical implications, as (1) cholesterol has to be synthesized de novo during embryonic development [2, 4]; (2) ARI is often prescribed to pregnant women [30–35]; 3) ARI is transported across the placenta and crosses the blood–brain barrier [36, 37]; (4) ARI disrupts the cholesterol biosynthesis and elevates 7-DHC levels in the toxic range [22–24, 28]; (5) 1–1.5% of the population carries a *DHCR7*^*+/*^^−^ mutation and might be more vulnerable to this medication. Based on these facts, we hypothesized that maternal ARI exposure will inhibit DHCR7 and increase 7-DHC in the developing brain of offspring, posing a serious risk to embryonic development. Furthermore, we proposed that embryos that carry a single-copy *DHCR7*^*+/*^^−^ mutation would show enhanced sensitivity to intrauterine ARI exposure. To test this hypothesis, we generated a time-pregnant mouse model where wild-type (WT) and *Dhcr7*^*+/*^^−^, carrying WT and *Dhcr7*^*+/*^^−^ embryos, respectively, were exposed to ARI or vehicle (VEH) from E12 to E19. The levels of cholesterol, its precursors, ARI, and its metabolites were measured at P0.

## Materials and methods

### Chemicals

Unless otherwise noted, all chemicals were purchased from Sigma-Aldrich Co (St. Louis, MO). High-performance liquid chromatographic-grade solvents were purchased from Thermo Fisher Scientific Inc. (Waltham, MA). Pharmaceutical-grade ARI (marketed as ABILIFY) was obtained from Bristol-Meyers and dissolved in ethanol for the experiments. All sterol standards, natural and isotopically labeled, used in this study are available from Kerafast, Inc. (Boston, MA).

### Mouse experiments

**Fig. 1:**
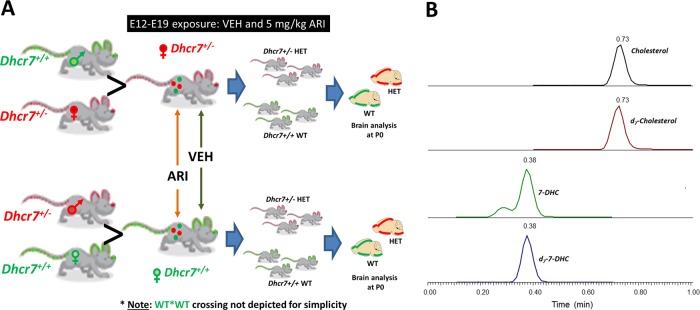
Experimental design. *Dhcr7*^*+/*^^−^ female mice were mated with wild-type (WT) males and *Dhcr7*^*+/*^^−^ male mice were mated with WT female mice (**a**). Pregnant mice were injected with either vehicle or 5 mg/kg aripiprazole from E12 to E19. At P0, pups were sacrificed and their brain sterol profile was analyzed. **b** denotes a typical liquid chromatography tandem mass spectrometric chromatogram denoting cholesterol, 7-dehydrocholesterol, and their respective internal standards

Adult male and female B6.129P2(Cg)-*Dhcr7*^*tm1Gst*^/J stock # 007453 mice were purchased from Jackson Laboratories. Mice homozygous for the *Dhcr7*^*Ex8*^ allele lack the exon 8 coding sequence and flanking splice acceptor site of the targeted gene, resulting in the truncated DHCR7 mutation most frequently observed in SLOS patients (IVS8-1G>C). Homozygous mice die shortly after birth [38, 39]. Heterozygous *Dhcr7*^*+/*^^−^ mice are well, fertile, and indistinguishable from control, WT mice. Mice were maintained by breeding within colony and refreshing twice a year with stock 000664 mice from Jackson Laboratories. The mice were housed under a 12 h light–dark cycle at constant temperature (25 °C) and humidity with *ad libitum* access to food (Teklad LM-485 Mouse/Rat Irradiated Diet 7912) and water in Comparative Medicine at UNMC, Omaha, NE. The breeding scheme and experimental design is denoted in Fig. 1. The time-pregnant female mice received intraperitoneal injections of VEH or ARI (5 mg/kg) from E12 to E19. Eight WT and eight *Dhcr7*^*+/*^^−^ mothers were used in our study. This exposure window was chosen based on the onset of cholesterol synthesis in the embryonic brain. It has been previously shown that the cholesterol biosynthesis in the brain starts at E12, and from this moment forward, the embryonic brain fully relies on its own cholesterol production [40]. Half of each genotype group was injected with VEH and the other half with ARI. The mouse colony was monitored three times a day and all newborn pups were collected for dissection shortly after the birth. Half of the cortex was used for sterol analysis and the other half was banked for follow-up experiments. Adult female mice were also sacrificed at the same time as pups. Ice-cold lysis buffer (120 mM NaCl, 50 mM HEPES, 1% Igepal) was added to frozen cortex samples and immediately sonicated. The total protein content was measured using BCA assay (Pierce) and used for normalization. All procedures were performed in accordance with the Guide for the Humane Use and Care of Laboratory Animals. The use of mice in this study was approved by the Institutional Animal Care and Use Committee of UNMC.

### Liquid chromatography tandem mass spectrometric (selective reaction monitoring (SRM)) analyses

After lysis, lipids were extracted and derivatized with PTAD as described previously [29] and placed in an Acquity UPLC system equipped with ANSI-compliant well plate holder coupled to a Thermo Scientific TSQ Quantis mass spectrometer equipped with an APCI source. Then 5 μL was injected onto the column (Phenomenex Luna Omega C18, 1.6 μm, 100 Å, 2.1 × 50 mm^2^) with 100% MeOH (0.1% v/v acetic acid) mobile phase for 1.0 min runtime at a flow rate of 500 μL/min. Natural sterols were analyzed by SRM using the following transitions: Chol 369 → 369, 7-DHC 560 → 365, desmosterol 592 → 560, lanosterol 634 → 602, with retention times of 0.7, 0.4, 0.3, and 0.3 min, respectively. SRMs for the internal standards were set to: d_7_-Chol 376 → 376, d_7_-7-DHC 567 → 372, ^13^C_3_-desmosterol 595 → 563, ^13^C_3_-lanosterol 637 → 605. Final sterol numbers are reported as nmol/mg of protein.

ARI levels were acquired in an Acquity UPLC system coupled to a Thermo Scientific TSQ Quantis mass spectrometer using an ESI source in the positive ion mode. Five μL of each sample was injected onto the column (Phenomenex Luna Omega C18, 1.6 μm, 100 Å, 2.1 × 50 mm^2^) using water (0.1% v/v acetic acid) (solvent A) and acetonitrile (0.1% v/v acetic acid) (solvent B) as mobile phase. The gradient was: 10–40% B for 0.5 min; 40–95% B for 0.4 min; 95% B for 1.5 min; 95–10% B for 0.1 min; 10% B for 0.5 min. ARI and its metabolites were analyzed by SRM using the following transitions: ARI 448 → 285, dehydroaripiprazole 446 → 285, 2,3-DCPP 230 → 187. The SRM for the internal standards (d_8_-ARI and d_8_-mCPP) were set to 456 → 293 and 204 → 157, respectively. Final drug levels are reported as ng/mg of protein.

### Statistical analyses

Statistical analyses were performed using Graphpad Prism 7 for Windows, Microsoft Excel and XLSTAT. Unpaired two-tailed *t* tests were performed for individual comparisons between two groups. The Welch’s correction was employed when the variances between the two groups was significantly different. *p* Values for statistically significant differences are highlighted in the figure legends. XLSTAT was used to do a comprehensive three-way analysis of variance (ANOVA) to assess the interaction between treatment (VEH vs ARI), maternal genotype (WT vs *Dhcr7*^*+/*^^−^) and embryonic genotype (WT vs *Dhcr7*^*+/*^^−^). The correlations between drugs were calculated using Pearson’s coefficient.

## Results

### ARI and its metabolites are detectable in the brains of treated pups

**Fig. 2:**
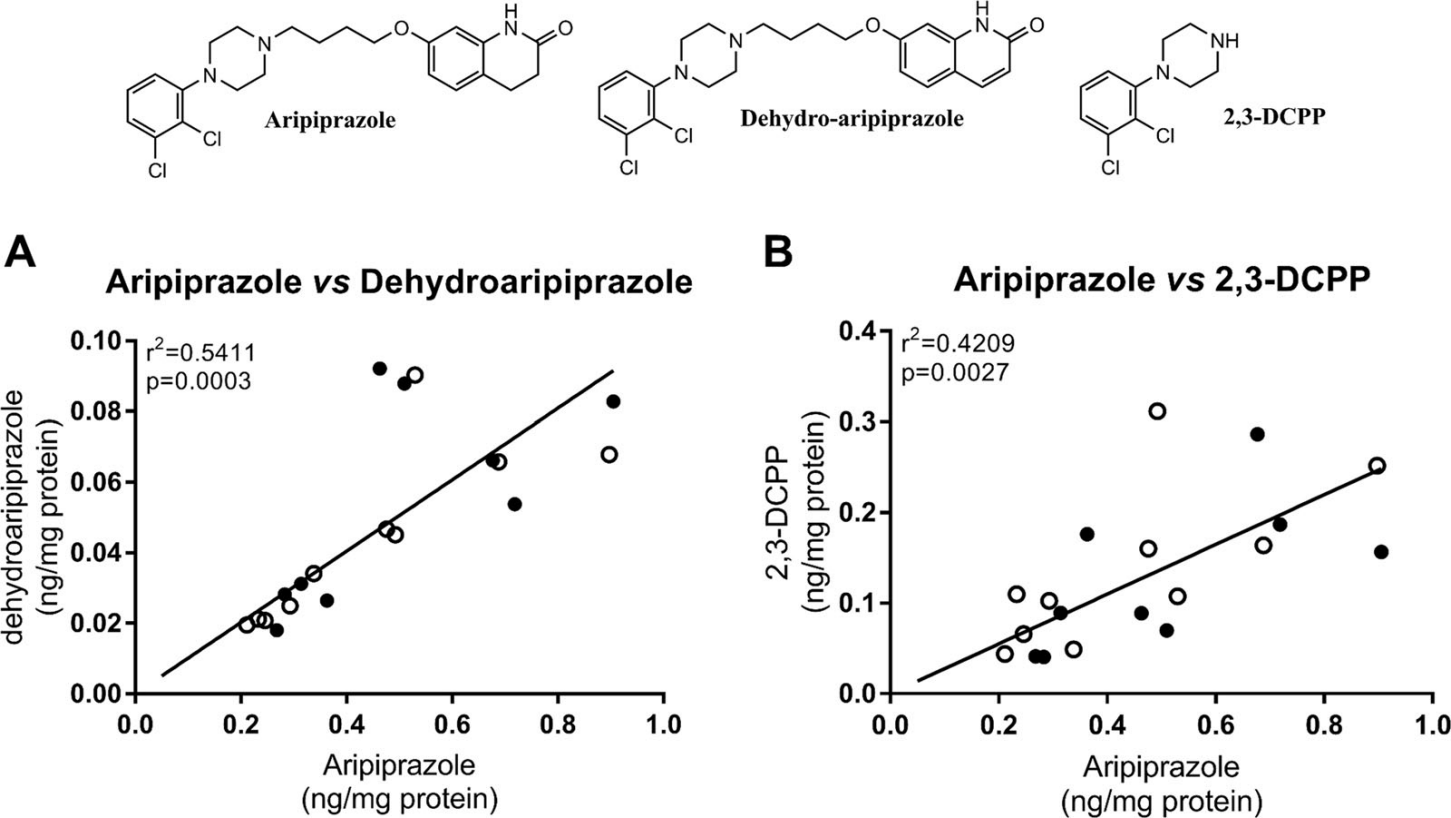
Aripiprazole (ARI) and its metabolites cross the placental barrier. ARI and its metabolites were readily detectable in the brain of newborn pups. A positive correlation between ARI and dehydroaripiprazole (**a**) and 2,3-dichlorophenylpiperazine (**b**) was observed in the analyzed tissue. Only ARI-treated pup data are disclosed, as in vehicle-treated pup brains no ARI or metabolites were detected. Each symbol denotes ARI level in a single ARI-treated brain sample (*n* = 19), filled symbols denote pups with a *Dhcr7*^*+/+*^ genotype (wild type), open symbols denote pups with a *Dhcr7*^*+/*^^−^ genotype. Pearson correlation coefficients (*r*^2^ and *p* values) were calculated using GraphPad Prism 7

To investigate the consequences of ARI to the offspring, time-pregnant WT and *Dhcr7*^*+/*^^−^ females were injected with either VEH or 5 mg/kg ARI. To confirm that maternal ARI exposure resulted in placental drug transfer to the embryos, we measured ARI and its metabolites in the brain of all tested pups. While none of the compounds were detected in the VEH-injected animals, ARI and its metabolites—dehydroaripiprazole and 1-(2,3-dichlorophenyl)piperazine (2,3-DCPP)—were detected in all brain samples from the ARI-injected group (Fig. 2). Importantly, we found a positive correlation between ARI and its metabolites in the brain of pups, regardless of maternal or embryonic genotype. This experiment confirmed that ARI was transported through the placenta, reached the brain of embryos, and had the potential to alter the sterol metabolism of the developing brain *in utero*.

### ARI decreases litter size

As an initial assessment of ARI’s impact on embryonic development, we examined the effect of ARI on the litter size and pups per litter. Table 1 shows the number of mothers used in each experimental group, number of pups delivered from each group, and the pups/mother ratio. The litter sizes from animals injected with ARI were substantially smaller when compared to those from animals injected with VEH: 57 pups were delivered from 8 females injected with VEH, while only 37 were delivered from the same number of females injected with ARI, representing a 35% reduction in the litter size (*p* < 0.05). This was also evidenced when the pups/mother ratio is analyzed, which was smaller in the groups that received ARI injection (VEH: 7.1 and ARI: 4.6 pups/mother). Moreover, while 3 stillborn pups were identified in the ARI-injected group, none were observed in the VEH-injected group. Interestingly, the mothers’ genotype (*Dhcr7*^*+/*^^−^ or *Dhcr7*^*+/+*^) had no apparent effect on these findings. Overall, these observations show that ARI has a considerable effect on offspring viability. While the smaller litters are clearly not a result of altered fertilization of embryonic implantation, we were unable the precisely determine the precise timing of the premature demise. Perhaps the three stillborn pups suggest that ARI exposure is likely to affect the later stages of pregnancy, but this should be further investigated. It is also noteworthy that we did not observe differences in the ratio of male-to-female pups in any of our treatment groups, suggesting that the effects of ARI are not preferentially affecting one of the sexes.

**Table 1:** Viability of VEH- and ARI-treated pregnancies

Treatment	Female (*n*)^a^	Pups		
		Total	(*Dhcr7*^*+/+*^)	(*Dhcr7*^*+/*^^−^)
Vehicle	*Dhcr7*^*+/+*^ (4)	26	15	11
	*Dhcr7*^*+/*^^−^ (4)	31	14	17
Aripiprazole^b^	*Dhcr7*^*+/+*^ (4)	17	8	9
	*Dhcr7*^*+/*^^−^ (4)	20	10	10

All VEH vs All ARI—*p* < 0.05

All *Dhcr7*^*+/+*^ vs All *Dhcr7*^*+/*^^*−*^—non-significant

VEH: *Dhcr7*^*+/+*^ vs *Dhcr7*^*+/*^^*−*^—non-significant

ARI: *Dhcr7*^*+/+*^ vs *Dhcr7*^*+/*^^*−*^—non-significant

*ARI* aripiprazole, *VEH* vehicle

^a^Each experimental group (*Dhcr7*^*+/+*^×VEH; *Dhcr7*^*+/*^^−^×VEH; *Dhcr7*^*+/+*^×ARI and *Dhcr7*^*+/*^^−^×ARI) consisted of four pregnant mice. Note that the ARI-treated litters were smaller (*Dhcr7*^*+/+*^—17 pups; *Dhcr7*^*+/*^^−^—20 pups) than the VEH-treated litters (*Dhcr7*^*+/+*^—26 pups, *Dhcr7*^*+/*^^−^—31 pups). Furthermore, ARI-treated litters resulted in three stillborn pregnancies. Note also that the pups/litter ratio is 35% smaller in the ARI-injected group (VEH: 7.1 pups/litter and ARI: 4.6 pups/litter). Of all the comparisons, only the VEH vs ARI pups/litter reached significance at *p* < 0.05. In addition, the sex of the pups did not differ between any of the compared groups

^b^Females were injected daily with either vehicle or 5 mg/kg aripiprazole from E12 to E19

### Pups born to *Dhcr7*^*+/*^^*−*^ mothers show increased vulnerability to ARI exposure

**Fig. 3:**
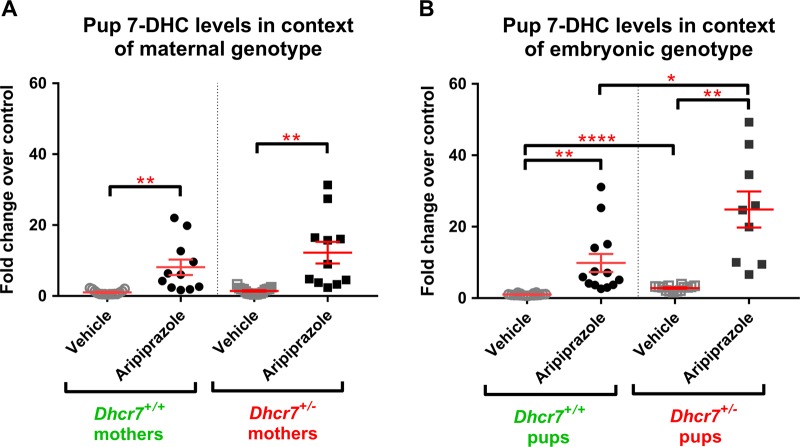
7-Dehydrocholesterol (7-DHC) levels in the P0 brain of pups maternally exposed to vehicle or aripiprazole (ARI) (5 mg/kg). **a** shows changes in 7-DHC in the context of the maternal genotype. Both wild-type (WT) and *Dhcr7*^*+/*^^−^ pups were grouped taking into account only their mothers’ genotype. **b** shows changes in 7-DHC in the context of the embryonic genotype. Pups were grouped taking into account only their own genotypes, regardless if they were born from WT or *Dhcr7*^*+/*^^−^ mothers. Note that 7-DHC is increased as a result of ARI injection and it is dependent both on the maternal (**a**) and embryonic genotypes (**b**). Values were normalized against the control condition (i.e., WT-Vehicle = 1). The genotypes are depicted below the groups; Statistical significance: *p < 0.05; **p < 0.01; ****p < 0.0001. Bars correspond to the mean ± SEM. Each symbol corresponds to a single pup brain. Note that *Dhcr7*^*+/*^^−^ pups have higher basal 7-DHC levels compared to their WT littermates (comparison between vehicle conditions in **b**), which becomes more pronounced in ARI-injected group. The raw sterol values are reported in Supplemental Material 1

Embryonic development is a period of active sterol biosynthesis in the brain [1, 2]. To assess the consequences of ARI treatment on the sterol profile during this period, we analyzed the brains of the pups immediately after birth (P0). To answer the question whether the maternal and embryonic genotypes affect the 7-DHC response to ARI, P0 pups were grouped and analyzed according to their mothers’ (Fig. 3a) or their own genotypes (Fig. 3b). 7-DHC values were normalized and are reported as fold change over control using VEH-injected pups from the WT group as the control condition (i.e., WT-vehicle = 1). We found that ARI induced a 12-fold increase in 7-DHC in the pups born to *Dhcr7*^*+/*^^−^ mothers, compared to an 8-fold increase in 7-DHC that was observed in mice from WT mothers (Fig. 3a). The more pronounced 7-DHC elevation in the brain of pups from *Dhcr7*^*+/*^^−^ mothers suggests that *Dhcr7*^*+/*^^−^ maternal genotype increases the vulnerability of pups to ARI exposure. Absolute 7-DHC levels are reported in Figure S1 in the supporting information. Levels of cholesterol (Figure S2), desmosterol (Figure S3), and lanosterol (Figure S4) were much less significantly changed and are included in the supporting information.

### ARI has the most pronounced biochemical effect on the brain of Dhcr7^+/^^−^ pups

We hypothesized that maternal ARI exposure would have the largest effect on 7-DHC in the brain of *Dhcr7*^*+/*^^−^ pups. Figure 3b denotes the changes in 7-DHC in response to ARI injection in the context of the embryonic *Dhcr7* genotype. Brain samples from WT pups injected with VEH had the lowest 7-DHC levels among all groups. *Dhcr7*^*+/*^^−^ pups had higher basal 7-DHC levels than their WT littermates, further suggesting mutations in one *Dhcr7* allele alone leads to an altered biochemical phenotype (comparison between VEH-exposed conditions). ARI increased 7-DHC levels in both WT and *Dhcr7*^*+/*^^−^ pups when compared to the VEH-injected groups, which confirms that ARI increases 7-DHC *in utero*. However, as hypothesized, *Dhcr7*^*+/*^^−^ pups were the most vulnerable to ARI’s effects and reported even higher 7-DHC levels than their WT littermates subjected to the same ARI exposure: while ARI injection resulted in a 10-fold increase in pups with a WT genotype, the same ARI exposure resulted in an even more remarkable, 25-fold increase in 7-DHC levels in pups with a *Dhcr7*^*+/*^^−^ genotype. These results indicate that a *Dhcr7*^*+/*^^−^ embryonic genotype also increases the vulnerability to ARI exposure. Altogether, the two panels of Fig. 3 clearly indicate that both maternal and embryonic *Dhcr7* genotypes are determinants of how the developing brain responds to ARI.

### Maternal genotype×embryonic genotype×ARI treatment interaction

**Fig. 4:**
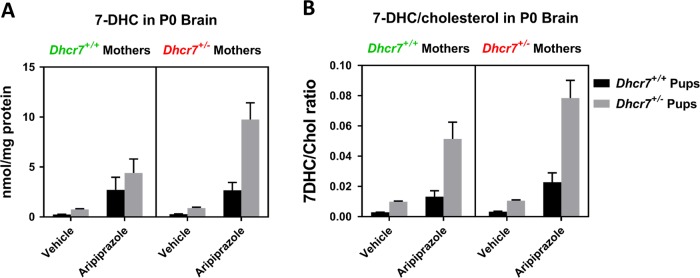
7-Dehydrocholesterol (7-DHC) levels found in the brain of P0 pups exposed to vehicle or aripiprazole (5 mg/kg). 7-DHC levels (**a**) and 7-DHC/cholesterol ratio (**b**) are grouped taking into account all the three variables: maternal genotype, embryonic genotype, and drug treatment (three-way analysis). Black and gray columns denote wild-type and *Dhcr7*^*+/*^^−^ pups, respectively. The mothers’ genotypes are depicted above the graph. Values correspond to the mean ± SEM. Note that the 7-DHC levels are the highest in *Dhcr7*^*+/*^^−^ pups from *Dhcr7*^*+/*^^−^ mothers, indicating a synergism between maternal genotype×embryonic genotype×treatment. Comprehensive statistical analysis of data is presented in Table 2

In the next step, we tested the hypothesis that the combination of both maternal and embryonic *Dhcr7*^*+/*^^−^ genotypes would alter ARI’s effect on 7-DHC, which would result in the highest and most toxic levels of 7-DHC. To address that, we did a three-way ANOVA analysis of 7-DHC levels in the brain of pups taking into account the maternal genotype (WT or *Dhcr7*^*+/*^^−^), the embryonic genotype (WT or *Dhcr7*^*+/*^^−^), and treatment (VEH or ARI). These results are presented in Fig. 4a and Table 2. As hypothesized, *Dhcr7*^*+/*^^−^ pups from *Dhcr7*^*+/*^^−^ mothers were the most vulnerable to ARI and presented the highest levels of 7-DHC. Figure 4a also shows that in all conditions *Dhcr7*^*+/*^^−^ pups had higher 7-DHC levels than their WT littermates. A comprehensive analysis of these results (Table 2) reveals that: (1) a combination of maternal and embryonic *Dhcr7*^*+/*^^−^ genotypes have a significant impact on 7-DHC; (2) ARI interacts with the maternal *Dhcr7*^*+/*^^−^ genotype and potentiates the effect on 7-DHC; (3) ARI interacts with the embryonic *Dhcr7*^*+/*^^−^ genotype and potentiates the effect on 7-DHC; (4) the most significant effect on 7-DHC comes from ARI treatment, which is further affected by both maternal and embryonic *Dhcr7*^*+/*^^−^ genotypes. Altogether, these results suggest that *Dhcr7*^*+/*^^−^ pups from *Dhcr7*^*+/*^^−^ mothers are highly vulnerable to ARI effects.

**Table 2:** Effect of three variables on 7-DHC levels in the pup brain

No.	Comparison	*p* Value
1	Treatment effect: ARI vs VEH	<0.0001
2	Mother genotype effect: *Dhcr7*^*+/*^^−^ vs Dhcr7^*+/+*^	0.010
3	Pup genotype effect: *Dhcr7*^*+/*^^−^ vs Dhcr7^*+/+*^	<0.0001
4	Two-way interaction: Treatment (ARI, VEH)×Maternal *Dhcr7* genotype (^*+/*^^−^*or* ^*+/+*^*)*	0.015
5	Two-way interaction: Treatment (ARI, VEH)×Pup *Dhcr7* genotype (^*+/*^^−^*or* ^*+/+*^*)*	<0.0001
6	Two-way interaction: Maternal *Dhcr7* genotype×Pup *Dhcr7* genotype	0.010
7	Three-way interaction: Treatment×Maternal *Dhcr7* genotype×Pup *Dhcr7* genotype	0.013

The table represents the outcome of multifactorial analysis of variance model progressively accounting for all three variables we tested: treatment (ARI, VEH), pup *Dhcr7* genotype (+/− vs +/+), and maternal *Dhcr7* genotype (+/− vs +/+). Rows 1–3 denote significance for each of the single variable; row 3–6 reports probability for two of the interacting factors; row 7 denotes interaction between all the three tested variables

*ARI* aripiprazole, *7-DHC* 7-dehydrocholesterol, *VEH* vehicle

Notably, these data are also concordant with our previously published data on the effect of ARI-treated *DHCR7*^*+/+*^ and *DHCR7*^*+/*^^−^ human dermal fibroblasts [22], suggesting that the *DHCR7 genotype*×*ARI treatment* interaction is not a mouse-specific physiological event, thus underscoring the potential clinical implications of our findings.

### ARI-induced alterations in the 7-DHC/cholesterol ratio are *Dhcr7* genotype-dependent

**Fig. 5:**
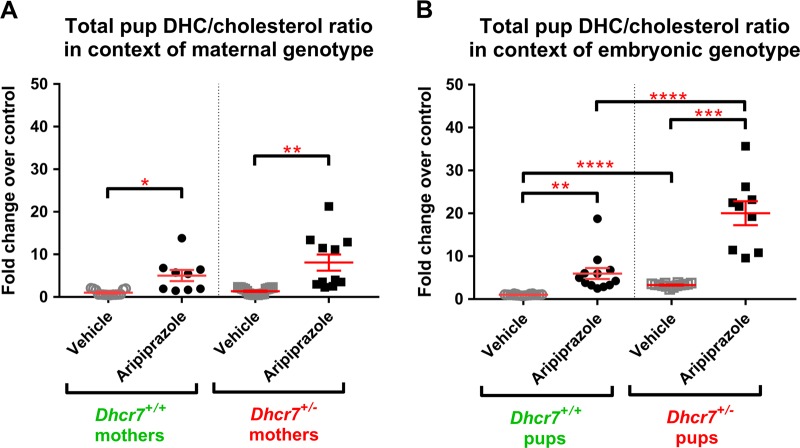
7-Dehydrocholesterol (7-DHC)/cholesterol ratio in the brain of P0 pups exposed to vehicle or aripiprazole (ARI; 5 mg/kg). **a** shows changes in 7-DHC/cholesterol in the context of the maternal genotype. Both wild-type (WT) and *Dhcr7*^*+/*^^−^ pups were grouped taking into account only their mothers’ genotype. **b** shows changes in 7-DHC/cholesterol in the context of the embryonic genotype. Pups were grouped taking into account only their own genotypes, regardless if they were born from WT or *Dhcr7*^*+/*^^−^ mothers. 7-DHC/cholesterol ratio is increased as a result of ARI injection and it is dependent on the maternal (**a**) and embryonic genotypes (**b**). Values were normalized against the control condition (i.e. WT-Vehicle = 1). The genotypes are depicted below the groups. Statistical significance: *p < 0.05; **p < 0.01; ***p < 0.001; ****p < 0.0001. Bars correspond to the mean ± SEM. Each symbol corresponds to a single pup. Note that the 7-DHC/cholesterol ratio in *Dhcr7*^*+/*^^−^ pups is elevated compared to their WT littermates (**b**). This difference is enhanced in the ARI-injected group, highlighting increased vulnerability of *Dhcr7*^*+/*^^−^ to ARI’s side effects. The raw sterol ratio values are reported in supporting information

In addition to the measurement of 7-DHC levels, we also assessed cholesterol biosynthesis, which can be measured by the 7-DHC/cholesterol ratio [22, 29]. Under control conditions, where cholesterol biosynthesis is functioning perfectly and the DHCR7 enzyme is operating properly, the 7-DHC/cholesterol ratio is extremely small (see Figure S5 in the supporting information for the “absolute” ratio values for all groups). DHCR7 inhibition leads to higher 7-DHC and lower cholesterol levels and therefore a higher 7-DHC/cholesterol ratio (Fig. 5), which can be used as a dynamic readout of the health of the sterol synthesis system.

Figure 5a denotes the ARI-induced changes in the 7-DHC/cholesterol ratio accounting for the mothers’ *Dhcr7* genotype. ARI induced a five-fold increase in the 7-DHC/cholesterol ratio in pups from WT mothers, while the same treatment lead to an eight-fold increase in the 7-DHC/cholesterol ratio in pups from *Dhcr7*^*+/*^^−^ mothers (Fig. 5a). These observations further suggest that a *Dhcr7*^*+/*^^−^ maternal genotype leads to an imbalance between cholesterol and its precursor.

ARI’s effects on the 7-DHC/cholesterol ratio in the context of pups’ genotype are shown in Fig. 5b. As observed previously for 7-DHC, the embryonic *Dhcr7*^*+/*^^−^ genotype had a pronounced effect both on the basal 7-DHC/cholesterol ratio and its response to ARI injection. Compared to the VEH-injected pups, the 7-DHC/cholesterol ratio was also significantly elevated in the ARI-injected pups. Indeed, while ARI injection increased the 7-DHC/cholesterol ratio by 6-fold in WT pups, the same treatment resulted in a 20-fold increase in the *Dhcr7*^*+/*^^−^ pup group, highlighting an imbalance of the sterol biosynthesis pathway.

An analysis of the 7-DHC/cholesterol ratio taking into account the maternal genotype, embryonic genotype, and treatment (three-way analysis) is presented in Fig. 4b. The 7-DHC/cholesterol ratio responded in a similar way as observed for 7-DHC in Fig. 4a, where the highest 7-DHC/cholesterol ratio is observed in *Dhcr7*^*+/*^^−^ pups from *Dhcr7*^*+/*^^−^ mothers injected with ARI. These results provide further evidence for a strong interaction between *maternal Dhcr7 genotype*×*embryonic Dhcr7 genotype*×*ARI treatment*.

## Discussion

Normal cholesterol metabolism is of critical importance for neurodevelopment. Once the blood–brain barrier is closed, which happens during embryonic development, the brain has to synthesize its own cholesterol [2, 4, 41]. The importance of a normal sterol metabolism is evidenced by the many genetic disorders associated with mutations in cholesterol biosynthesis enzymes [5–8]. Therefore, a chemical interference with this biosynthetic pathway could have profound effects on the development and health of the offspring.

ARI has been used to treat patients with schizophrenia and bipolar disorders for many years and its beneficial effects to patients are well known [42, 43]. Side effects of ARI have been extensively documented, including the modulation of the cholesterol biosynthesis both in vitro and in vivo [23, 28, 42], but relatively little attention has been paid to these processes in the context of pregnancy, maternal and offspring genotype, and potential pathological changes in the offspring.

Our results show that maternal exposure to ARI is potentially deleterious on embryonic development. There are eight major conclusions that we can draw from our study. (1) ARI and its metabolites are transported across the placenta and reach the brain of fetuses during embryonic development. (2) VEH-treated *Dhcr7*^*+/*^^−^ pups have higher than normal 7-DHC levels than their WT littermates, which is in concordance with observations in adult humans carrying the *DHCR7*^*+/*^^−^ genotype. (3) Maternal ARI exposure leads to decreased viability of embryos, as evidenced by reduced litter size. (4) ARI inhibits the DHCR7 enzyme in the brain of all embryos and leads to increased 7-DHC levels, regardless of maternal or offspring *Dhcr7* genotypes. (5) ARI increases 7-DHC levels in WT pups to levels higher than those observed in *Dhcr7*^*+/*^^−^ pups under VEH-treated conditions. (6) Based on the sterol biosynthesis profile, *Dhcr7*^*+/*^^−^ pups are more vulnerable to maternal ARI exposure than their WT littermates. This *drug×genotype* interaction might lead to a chemically induced SLOS phenotype, which should be further investigated. (7) There is a significant difference between the response of the pups from WT and *Dhcr7*^*+/*^^−^ mothers to maternal ARI exposure, and we can conclude that the mothers’ genotype also influences the pups’ response to the treatments and increases their vulnerability to cholesterol biosynthesis inhibitors. (8) Based on 7-DHC level measurements, maternal *Dhcr7*^*+/*^^−^ genotype, embryonic *Dhcr7*^*+/*^^−^ genotype, and ARI treatment interact and potentiate each other’s effects. This suggests that both mothers’ and pups’ genotype carry the vulnerability to ARI exposure. This confirms our initial hypothesis that the strongest effects of maternal ARI exposure would be observed in *Dhcr7*^*+/*^^−^ pups originating from *Dhcr7*^*+/*^^−^ pregnant dams. This simultaneously highlights that paternally inherited *DHCR7*^*+/*^^−^ genotype combined with ARI exposure represents a risk, even when the mother is *DHCR7*^*+/+*^. This should be examined in follow-up studies.

So, the first critical question becomes: what are the overall biological consequences of 7-DHC elevation and disruption of sterol biosynthesis observed in our studies? Our study did not assess the gross anatomical and microanatomical brain changes in the ARI-exposed pups or attempted behavioral assessments. Based on changes in the brains of patients with SLOS and animal models of the disorder, we can only hypothesize at this time that observed biochemical impact would give rise to alteration in the serotonergic system [44] and corpus callosum connectivity [45], and this should be further investigated. Still, several recent publications indicate that our findings should not be taken lightly. Recently, Bolland and Tatonetti comprehensively reviewed the effects of cholesterol-altering drugs on pregnancy outcomes in humans [46] and linked inhibitors of cholesterol biosynthesis to a wide range of negative pregnancy outcomes. Importantly, they found that antipsychotics, such as clozapine and haloperidol and cholesterol-lowering drugs (statins), were among those with the most deleterious effects on fetal development. Notably, these findings did not account for a *DHCR7* genotype as a variable that might further influence the outcome. Thus these findings suggest that any pharmacologically active substance should be closely examined for its potential to interfere with offspring cholesterol biosynthesis during pregnancy, and perhaps it is worth considering a dual parental and fetal DHCR7 genotyping before prescribing such medications.

If we accept that there are potential biological consequences of unwanted 7-DHC elevation, it leads us to the second question: should pharmacological compounds with effects on cholesterol biosynthesis be prescribed to *DHCR7*^*+/*^^−^ mutation carrier children and adults? The answer to this question is less clear: while *DHCR7*^*+/*^^−^ mutation carriers have elevated baseline DHC levels, they are generally considered healthy, and there is no current data that speak of drug×genotype interactions in these individuals. Clearly, this could be examined in epidemiological genome-wide association studies. However, it is clear that individuals with SLOS, who already carry two mutant *DHCR7* alleles and have remarkable elevations of 7-DHC, should not be prescribed ARI and other medications that act as cholesterol biosynthesis inhibitors.

In conclusion, considering that ARI is a very commonly used medication, often prescribed to pregnant women, we believe that there are important clinical implications of our study. (1) SLOS patients should clearly avoid drugs that increase 7-DHC levels, as they have already extremely high, toxic levels of 7-DHC, and any further increase might be detrimental. (2) Treatment with 7-DHC-elevating substances (such as ARI, trazodone, and haloperidol) might be potentially unsafe for the 1–1.5% of population with single-allele disruptions of the *DHCR7* gene. (3) Vulnerability to 7-DHC-elevating compounds appears to be most pronounced during pregnancy and brain development, and both prenatal and parental genetic testing for *DHCR7* should be considered before prescribing sterol-interfering medications during pregnancy. Such testing or a choice of a different medication could avoid a chemically induced SLOS phenotype in offspring who carries a single allele disruption of the *DHCR7* gene. Finally, it should be stressed that ARI (and many other medications affecting sterol biosynthesis) are safe and life-saving medications for 99% of the population, but in this era of precision/personalized medicine, we must recognize the potential vulnerability of a subpopulation of our patients to these pharmacological compounds. After all, this *maternal genotype*×*embryonic genotype*×*treatment* interaction is a cornerstone of personalized medicine and moves us from the *primum non nocere* to the *primum non nocere et optimum curare* concept.

## Supplementary information

Supplemental Material
