# Supplementary material for: Maternal aripiprazole exposure interacts with 7-dehydrocholesterol reductase mutations and alters embryonic neurodevelopment
Source: Mol Psychiatry. 2019 Feb 11;24(4):491–500. doi: 10.1038/s41380-019-0368-6 (PMC6477890; doi:10.1038/s41380-019-0368-6)
Supplement: Supplementary file 1 — Supplemental Material [file 41380_2019_368_MOESM1_ESM.doc]

**Supplemental information**

Maternal aripiprazole exposure interacts with 7-dehydrocholesterol reductase mutations and alters embryonic neurodevelopment

Thiago C. Genaro-Mattos^1^, Luke B. Allen^2^, Allison Anderson^1^, Keri A. Tallman^3^, Ned A. Porter^3^, Zeljka Korade^2^ and Károly Mirnics^1*^

^1^Munroe-Meyer Institute, University of Nebraska Medical Center, Omaha, NE

^2^Department of Pediatrics, University of Nebraska Medical Center, Omaha, NE

^3^Department of Chemistry, Vanderbilt University, Nashville, TN.

**SUMMARY**

Scheme 1…………………………………………………………………………………..**Page S2**

Supplemental Figure 1……………………………………………………………………..**Page S3**

Supplemental Figure 2……………………………………………………………………..**Page S4**

Supplemental Figure 3……………………………………………………………………..**Page S5**

Supplemental Figure 4……………………………………………………………………..**Page S6**

Supplemental Figure 5……………………………………………………………………..**Page S7**

**Scheme 1**


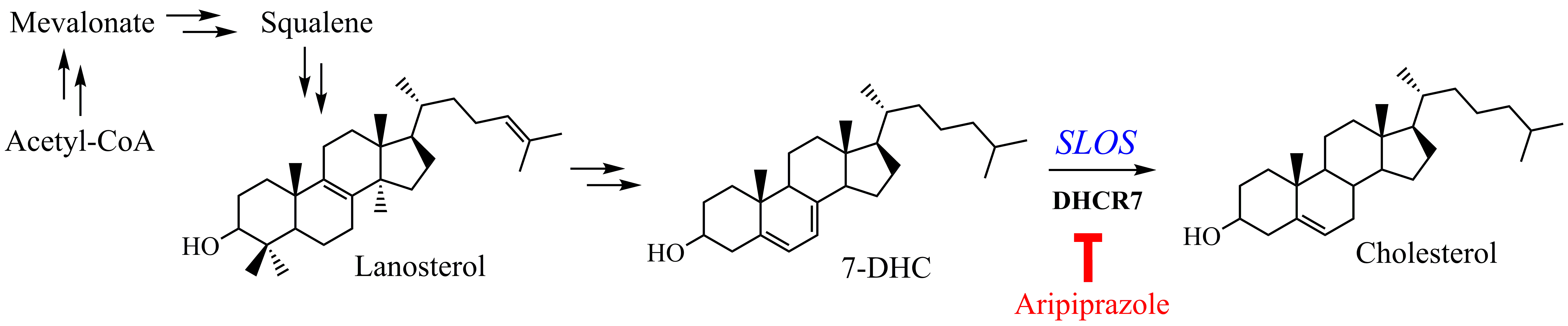


**Scheme 1.** Simplified cholesterol biosynthesis pathway highlighting the conversion of 7-DHC into cholesterol by the enzyme DHCR7. Mutations in this enzyme lead to the neurodevelopmental disorder known as SLOS. Antipsychotics, such as aripiprazole, inhibit the DHCR7 enzyme and leads to a similar biochemical phenotype, which is characterized by increased 7-DHC levels.

**Supplemental Figure 1**


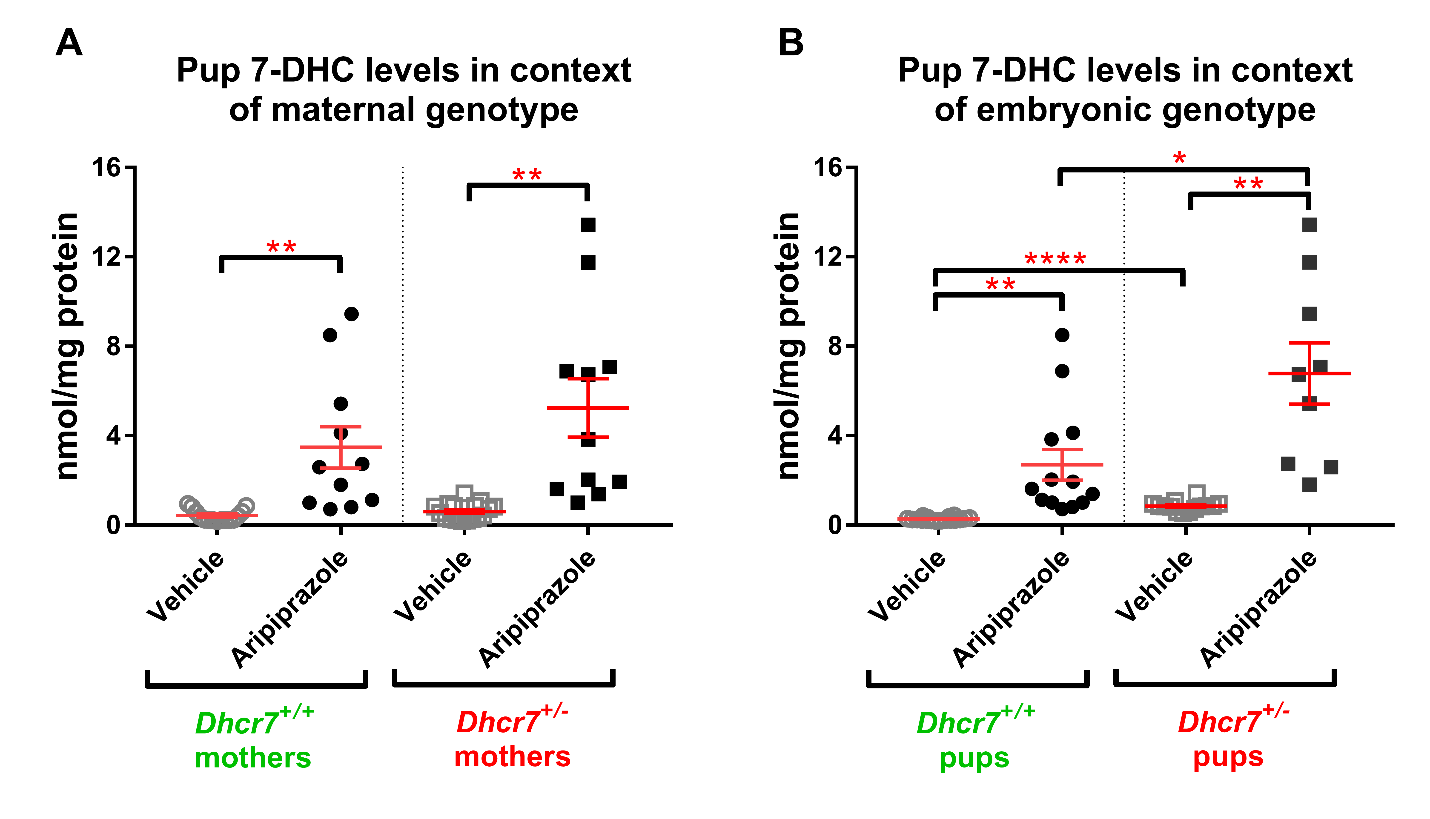


**Supplemental figure 1**. 7-**DHC levels in the brain of P0 pups exposed to vehicle or ARI (5mg/kg)**. Values correspond to the absolute 7-DHC levels detected in the brain (nmol/mg protein). This figure is a different representation of the result presented in **Figure 4** in the main manuscript. Statistical significance: **p*<0.05; ***p*<0.01; *****p*<0.0001. Bars correspond to the mean ± SEM.

**Supplemental Figure 2**


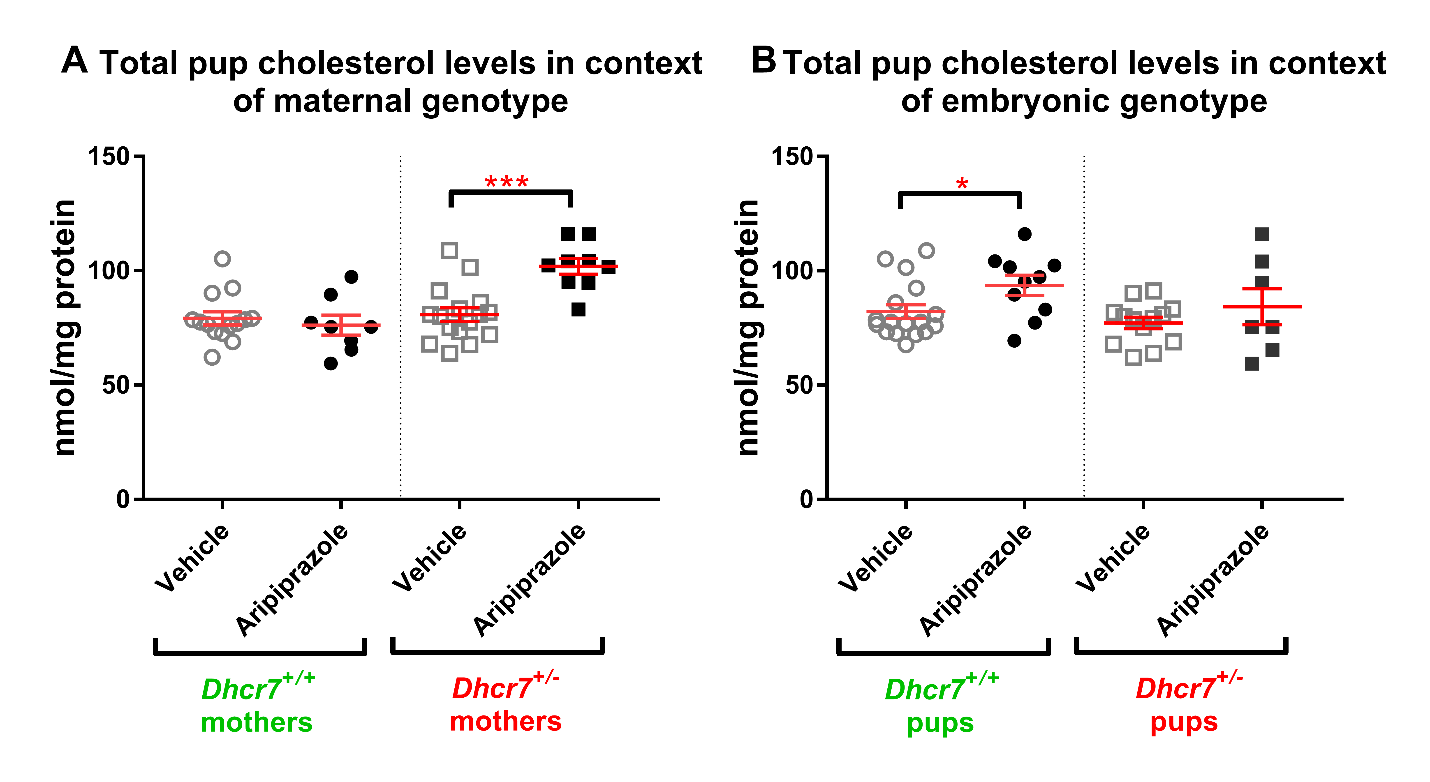


**Supplemental figure 2**. **Cholesterol levels in the brain of P0 pups exposed to vehicle or ARI (5mg/kg)**. **Panel A** shows changes in cholesterol levels in the context of the maternal genotype. Both WT and *Dhcr7^+/-^* pups were grouped by their mothers’ genotype. **Panel B** shows changes in cholesterol in the context of the embryonic genotype. Pups were grouped based on their own genotypes, regardless if they were born from WT or *Dhcr7^+/-^* mothers. The genotypes are depicted below the groups; Statistical significance: **p*<0.05; ****p*<0.001; Bars correspond to the mean ± SEM. Each symbol corresponds to a single pup brain. Values correspond to the absolute cholesterol levels detected in the brain (nmol/mg protein).

**Supplemental Figure 3**


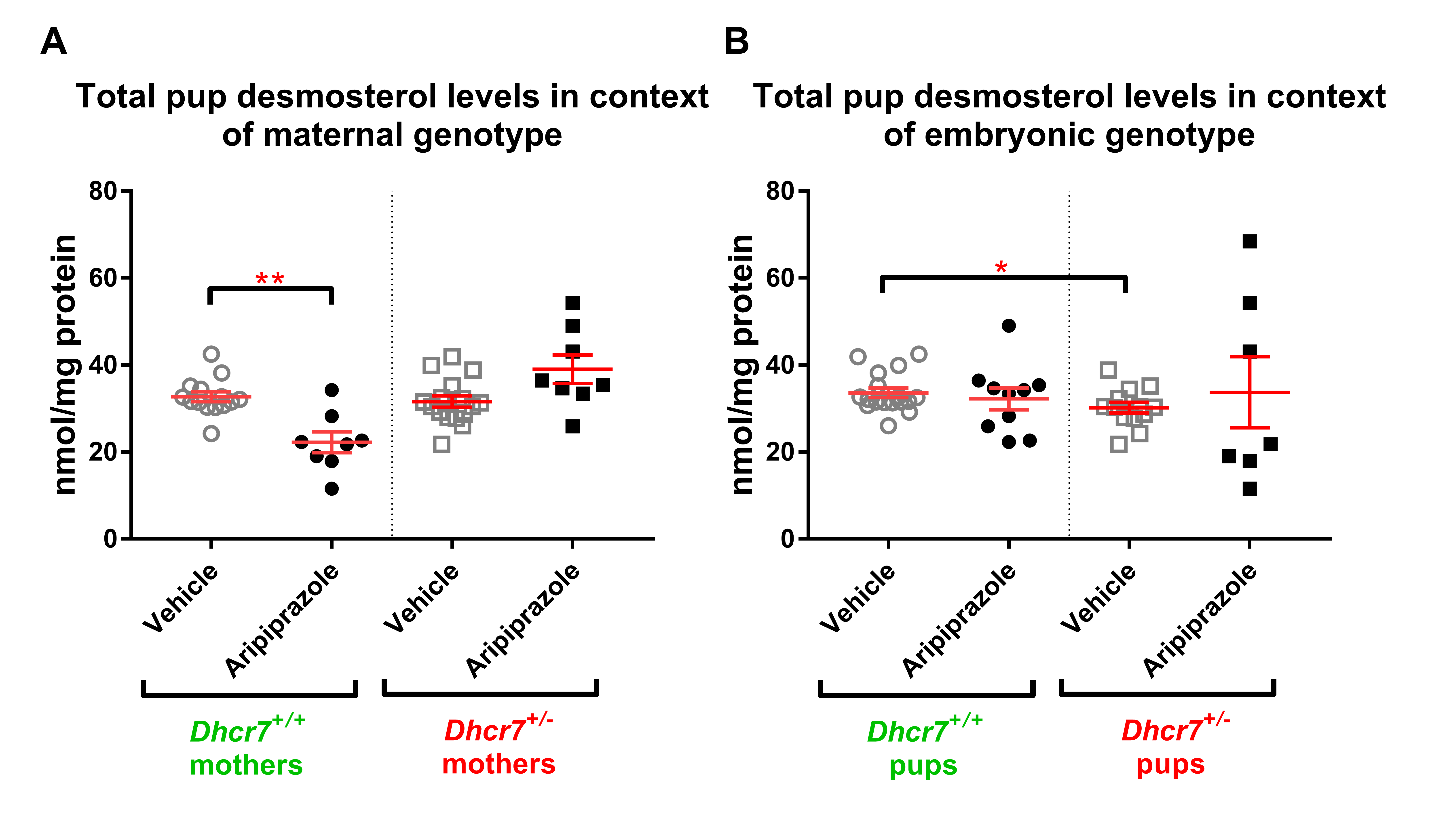


**Supplemental figure 3**. **Desmosterol levels in the brain of P0 pups exposed to vehicle or ARI (5mg/kg)**. **Panel A** shows changes in desmosterol in the context of the maternal genotype. Both WT and *Dhcr7^+/-^* pups were grouped by their mothers’ genotype. **Panel B** shows changes in desmosterol in the context of the embryonic genotype. Pups were grouped based on their own genotypes, regardless if they were born from WT or *Dhcr7^+/-^* mothers. The genotypes are depicted below the groups; Statistical significance: **p*<0.05; ***p*<0.01; Bars correspond to the mean ± SEM. Each symbol corresponds to a single pup brain. Values correspond to the absolute desmosterol levels detected in the brain (nmol/mg protein).

**Supplemental Figure 4**


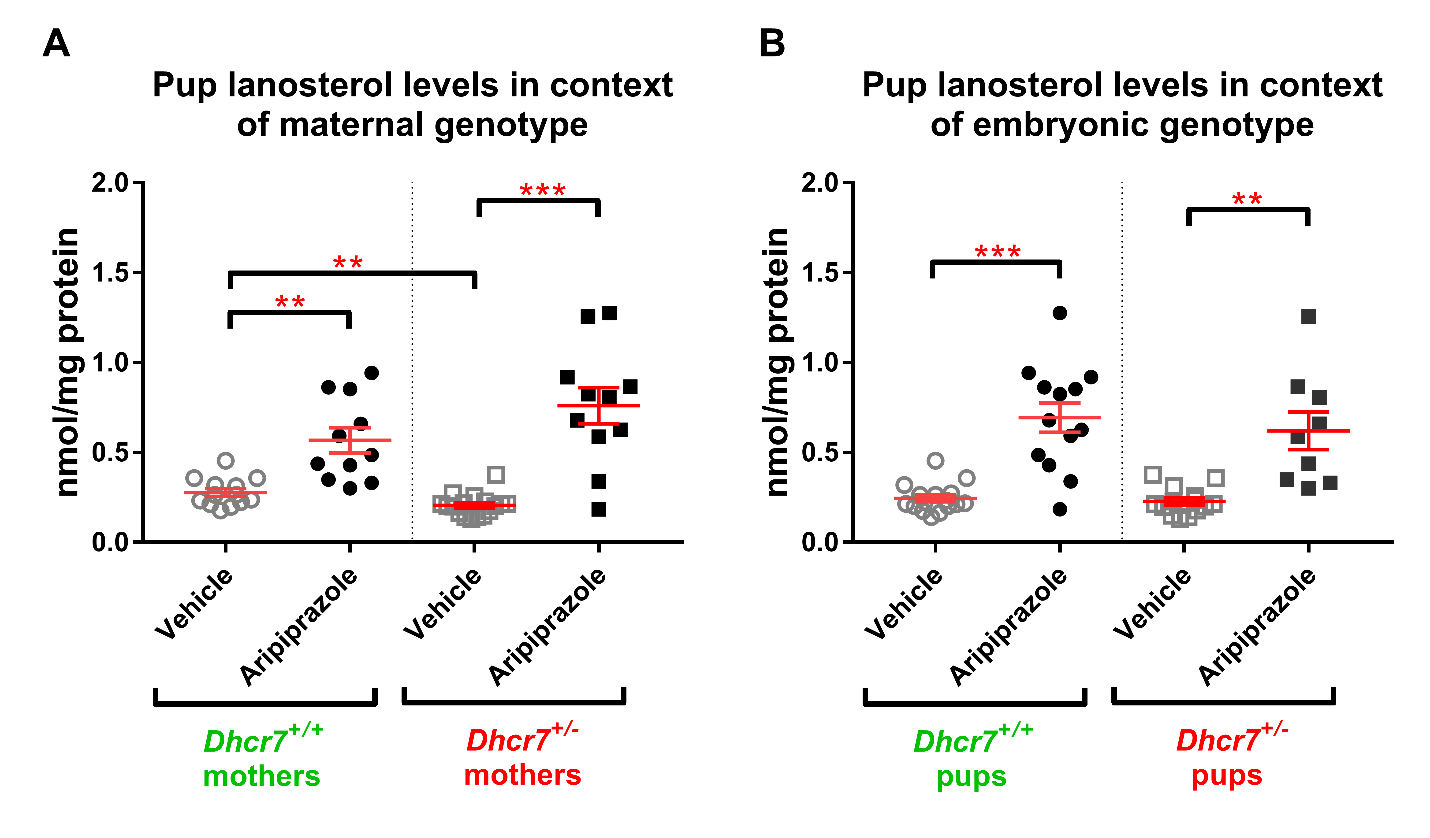


**Supplemental figure 4**. **Lanosterol levels in the brain of P0 pups exposed to vehicle or ARI (5mg/kg)**. **Panel A** shows changes in lanosterol in the context of the maternal genotype. Both WT and *Dhcr7^+/-^* pups were grouped by their mothers’ genotype. **Panel B** shows changes in lanosterol in the context of the embryonic genotype. Pups were grouped based on their own genotypes, regardless if they were born from WT or *Dhcr7^+/-^* mothers. The genotypes are depicted below the groups; Statistical significance: ***p*<0.01; ****p*<0.001; Bars correspond to the mean ± SEM. Each symbol corresponds to a single pup brain. Values correspond to the absolute lanosterol levels detected in the brain (nmol/mg protein).

**Supplemental Figure 5**


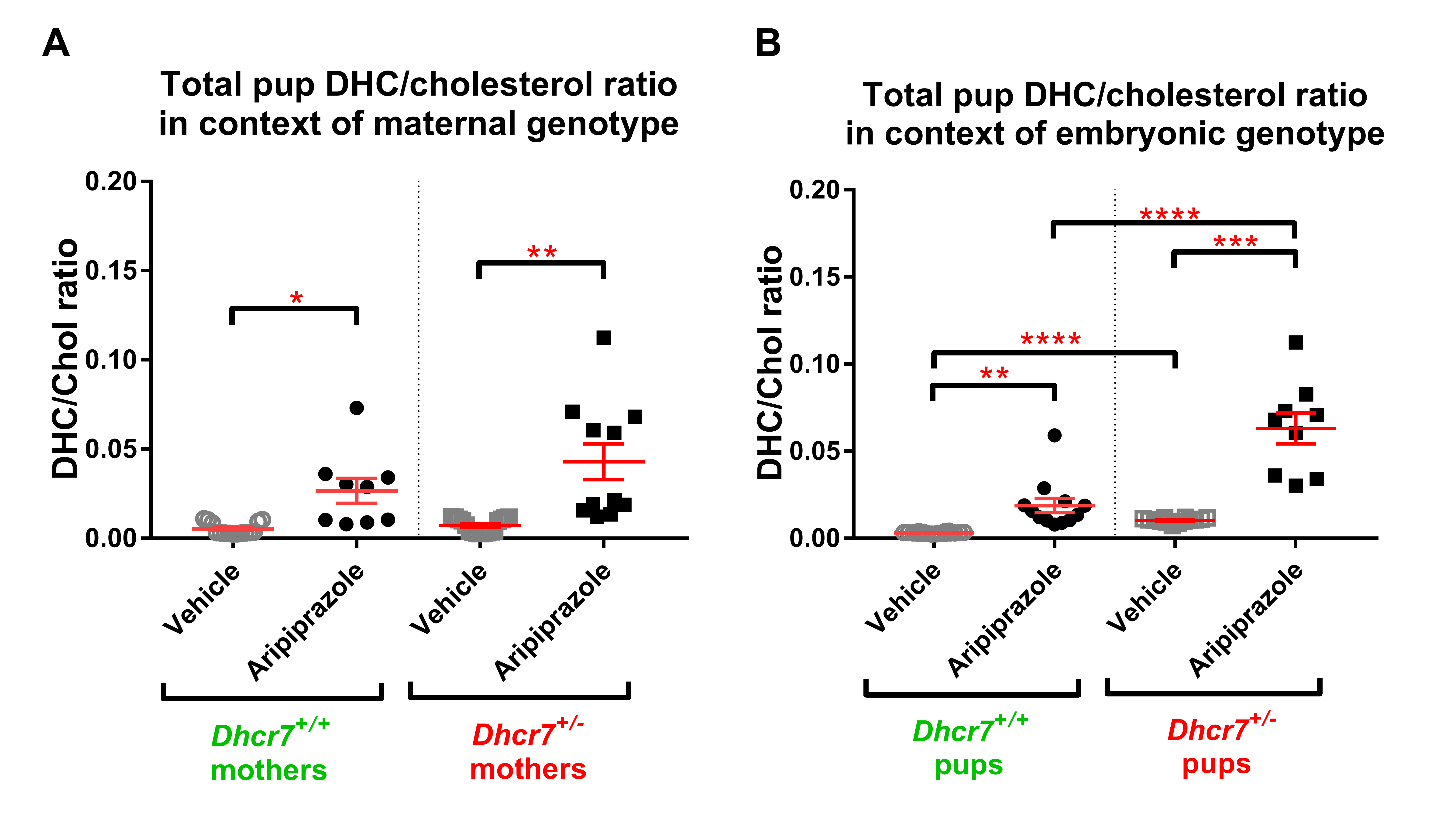


**Supplemental figure 5**. 7-**DHC/cholesterol ratio of sterols detected in the brain of P0 pups exposed to vehicle or ARI (5mg/kg)**. **Panel A** shows changes in the 7-DHC/cholesterol ratio in the context of the maternal genotype. Both WT and *Dhcr7^+/-^* pups were grouped by their mothers’ genotype. **Panel B** shows changes in the 7-DHC/cholesterol ratio in the context of the embryonic genotype. Pups were grouped based on their own genotypes, regardless if they were born from WT or *Dhcr7^+/-^* mothers. Bars correspond to the mean ± SEM. Each symbol corresponds to a single pup brain. This figure is a different representation of the result presented in **Figure 6** in the main manuscript. Statistical significance: **p*<0.05; ***p*<0.01; ****p*<0.001; *****p*<0.0001. Bars correspond to the mean ± SEM.
